# Early biochemical effects of velmanase alfa in a 7‐month‐old infant with alpha‐mannosidosis

**DOI:** 10.1002/jmd2.12144

**Published:** 2020-07-10

**Authors:** Lucia Santoro, Lucia Zampini, Lucia Padella, Chiara Monachesi, Stefania Zampieri, Andrea Dardis, Rosanna Cordiali, Tiziana Galeazzi, Carlo Catassi

**Affiliations:** ^1^ Department of Clinical Sciences, Division of Pediatrics Polytechnic University of Marche, Ospedali Riuniti, Presidio Salesi Ancona Italy; ^2^ Regional Coordinator Centre for Rare Diseases Academic Hospital "Santa Maria della Misericordia" Udine Italy

**Keywords:** alpha mannosidosis, enzyme replacement therapy, Hematopoietic cell transplantation, liquid chromatography coupled with tandem mass spectrometry, oligosaccharides, thin layer chromatography

## Abstract

Alpha mannosidosis is an ultrarare pathology with variable phenotypic manifestations, characterized by the deficiency of lysosomal alpha mannosidase which causes accumulation of neutral oligosaccharides. Until recently, the hematopoietic stem cell transplantation was the only clinical feasible therapeutic option. Only in 2018, the European Medicines Agency's committee approved the recombinant enzyme velmanase alfa for long‐term treatment of non‐neurological manifestations in mild and moderate forms of alpha‐mannosidosis. In this study, the very early biochemical effects of enzyme replacement therapy in in a 7‐month‐old patient with alpha‐mannosidosis were described. Velmanase alpha was administered as supporting therapy awaiting for hematopoietic stem cell transplantation, the treatment chosen for the patient because of the early onset form. The results showed that the enzyme replacement therapy was able to reduce the content of three different mannosyl‐oligosaccharides monitored by tandem mass spectrometry after 2 months of treatment. In particular, the mean relative changes from baseline values were −67% in urine and −53% in serum at the latest observation. The study also showed that the enzymatic activity detected in serum 1 week after the first infusion was four times higher than the normal values and constant in the following points of observation. These findings led us to assume that velmanase alfa might be biologically active in this young patient.

**Synopsis**

Enzyme replacement therapy in a 7 months old patient with alpha‐mannosidosis was effective in ensuring the presence of significant enzyme activity and reducing stored mannosyl‐oligosaccharides during 2 months of treatment.

**Abbreviations**
ERT: enzyme replacement therapy
HSCT: hematopoietic cell transplantation
LC‐MS/MS: liquid chromatography coupled with tandem mass spectrometry
MRM: multiple reactions monitoring
OS: oligosaccharides
TLC: thin layer chromatography
VA: Velmanase alfa

## 1 INTRODUCTION

Alpha‐mannosidosis is an ultra‐rare condition with a prevalence estimated at 1:250 000 to 1:1 000 000 live births.^1^ It is an autosomal recessive, multisystemic, progressive lysosomal storage disorder caused by alpha‐mannosidase deficiency. Impaired enzyme activity leads to abnormal and widespread accumulation of unprocessed oligosaccharides (OS), causing progressive damage including facial and skeletal abnormalities, motor and cognitive impairment, hearing loss and immune deficiency. Clinical subtypes may range from mild to severe. Mild forms are slowly progressing with attenuated symptoms generally diagnosed after the first decade of life, moderate forms can be diagnosed in preschool age, while severe forms show rapid progression and may manifest as prenatal loss or early death due to neurological involvement.^2^ Nonetheless, recent literature has suggested considering alpha‐mannosidosis as a continuum of symptoms.^3^, ^4^, ^5^

So far, 155 pathogenic variants in the gene *MAN2B1* associated with alpha‐mannosidosis have been described worldwide but there is no clear correlation between genotype and phenotype.^5^

Until recently, the hematopoietic stem cell transplantation (HSCT) has been the sole therapeutic option available. Early transplantation is generally recommended as a leading therapeutic option to prevent most of the systemic complications, stabilize or even improve skeletal abnormalities and prevent the cognitive decline and early death.^5^, ^6^ Only in 2018, the European Medicines Agency's committee approved the recombinant enzyme velmanase alfa (VA) for long‐term treatment of non‐neurological manifestations in mild and moderate forms of alpha‐mannosidosis.^6^ In a prospective study of long‐term efficacy and safety of VA enzyme replacement therapy (ERT) on 25 affected subjects (aged 6‐35 years), were observed significant improvements in serum OS levels and in functional parameters such as endurance, pulmonary function, and motor proficiency after an average of 29.3 months of treatment.^7^, ^8^

The first clinical trial investigating the safety and efficacy of repeated VA treatment in pediatric patients below 6 years of age (presently at Phase 2) started in 2016 and is estimated to be completed in 2020 (ClinicalTrials.gov Identifier: NCT02998879).

In this report are showed the effects of VA treatment applied for the first time in a 7 months old patient with alpha‐mannosidosis and monitored through mannosyl‐oligosaccharides (OS) content and enzymatic activity.

## 2 PATIENT REPORT

We describe the case of a 5‐month‐old male infant, first child of a non‐consanguineous couple, born at 37^3/7^ weeks gestational age, following an uneventful pregnancy and eutocic delivery, with a birth weight of 3650 g, birth length of 51 cm and neonatal physiologic jaundice. Neurodevelopmental milestones: first smile at one and a half month and head control acquired at 2 to 3 months. No allergies or infectious diseases, often rhinitis and snoring. The patient was initially referred to the Metabolic Centre of Salesi Children's Hospital for evaluation of a dorsolumbar gibbus, observed by his primary care physician. On admission, physical examinations revealed prominent forehead, bilateral hydrocele, moderate right inguinal hernia and moderate hepatomegaly and splenomegaly. Radiologic examination revealed reduced bone mineral density and the characteristic pattern of dysostosis multiplex. The neurological evaluation showed abundant sialorrhea and mild hypotonia with normal psychomotor development.

The initial laboratory findings in urine showed the presence of mannosyl‐OS by LC‐MS/MS and the almost absent activity of lysosomal alpha‐mannosidase in leucocytes (0.1% residual activity) confirmed the diagnosis of alpha‐mannosidosis.

The genetic analysis identified two so far undescribed mutations in MAN2B: c.1468_1472 del TTCAC (p.F490Lfs*25)] + [c.1229A > C (p.Q410P), one small deletion causing a shifting of the reading frame probably leading to the synthesis of a truncated protein, and a missense variant leading to the synthesis of an almost completely inactive enzyme (data not shown).

Immediately after the diagnosis, based on clinical, biochemical and molecular findings the procedures for HSCT were planned and after the ethical committee approval for compassionate use, the patient was started on intravenous ERT with VA at 7 months old as supporting therapy waiting for HSCT. The infusions were administered in the Paediatric Clinic of Salesi Children Hospital every week at the dose of 1 mg/kg of body weight. Each infusion lasted 50 minutes and was preceded by premedication with paracetamol and antihistamine.

## 3 MATERIALS AND METHODS

### 3.1 Reagents

Maltoheptaose (Glc7) was from Sigma‐Aldrich (Saint Louis, MO). Acetonitrile for LC‐MS/MS and formic acid were from Merck (Darmstadt, Germania). Ultrapure water was from Easypure Rodi (Milano, Italy).

### 3.2 Patient sample collection

Blood and urine samples were collected before VA infusions for 2 months at alternative weeks.

After blood centrifugation, serum and urine samples were stored at −20°C until analysis.

### 3.3 Isolation and separation of mannose‐OS by thin layer chromatography

Neutral OS extracted from equal aliquots of urine and serum before and after 8 weeks of treatment were loaded onto TLC plates (20 × 20 Silica gel 60F, Merck), and the separation was performed as the method described by Roces et al.^9^

### 3.4 LC‐MS/MS analysis of mannosyl‐OS

The LC‐MS/MS method for determination of urinary mannosyl‐OS as described by Piraud et al^10^ was used for the analysis of urine samples. This method was adapted to determine mannosyl‐OS also in serum samples: a 100 μL volume of serum was mixed with 10 μL of Glc7 IS (100 mg/L) and 100 μL of 100% Acetonitrile. After centrifugation, 100 μL of supernatant were mixed with 100 μL Acetonitrile 37.5% and 10 μL were injected for analysis.

Liquid chromatography was developed on a NH2 column (Luna 3 μm, 100 Å, 50 mm × 2.1 mm, (Torrence, USA) maintained at 40°C, applying a flow rate of 400 μL/min under an elution gradient of solvents A (formic acid 0.2% [v/v] in H2O) and B (formic acid 0.2% [v/v] in Acetonitrile): 85% B for 0.2 minute; from 85% to 50% B in 3.8 minutes; from 50% to 5% B in 1 minute; 5% B during 15 minutes; from 5% to 85% B in 1 minute and re‐equilibration time with 85% B up 26 minutes.

The MS/MS analysis was performed on a Agilent 6410 Triple Quadruple in positive ionization mode, with 4.0 kV capillary voltage, 25 psi nebulizer, and gas flow to 10 L/min at 300°C. Data analysis was conducted using the software MassHunter Workstation and the Quantitative Analysis software (version B.07.00). Ratios of peak area/IS area were calculated for each multiple reaction monitoring (MRM). Results were expressed as multiple of the median of the unaffected patients.

Eight MRM from three different mannosyl‐OS were monitored, including six different product ions to detect the mannosyl‐oligosaccharides type I (Mann1‐OS) with *m/z* 568.2:244.1, 347.2, 365.2, 406.3, 467.3, 550.3, and two specific transitions (730.3 > 509.3 and 892.4 > 671.4) for Mann2‐OS and Mann3‐OS, respectively.

### 3.5 Enzymatic assays

Alpha‐mannosidase activity in serum and leucocyte samples was assayed incubating for 1 hour at 37°C with 4‐methylumbelliferyl‐alpha‐d‐mannopyranoside, at pH 4.0. Fluorescence was determined with a fluorometer at 365 (excitation) and 446 nm (emission) and the enzyme activity was expressed as nmol/mL/h for serum and as nmol/mg protein/h for leucocytes.^11^

## 4 RESULTS

### 4.1 Patient clinical evaluation

From a clinical point of view there were no infusion‐related reactions during ERT treatment. At follow‐up visit after eight weekly infusions (2 months under ERT), the child presented with anthropometric measurement within the normal ranges, same coarsened facial features, improved snoring, mild neurological involvement, no alterations at audiometry and eye examinations. Signs of dysostosis multiplex and aortic valve insufficiency were not reassessed during that follow‐up visit due to the short time interval. By ultrasound, a slight reduction of hepatomegaly and a stabilization of splenomegaly were observed.

### 4.2 Mannosyl‐OS determination

The TLC of the urine sample collected before ERT showed the migration of eight bands under lactose. As reported by Roces et al,^9^ they should correspond to mannosyl‐OS composed of 2 to 9 mannose residues and a single *N*‐acetylglucosamine residue.

**FIGURE 1:**
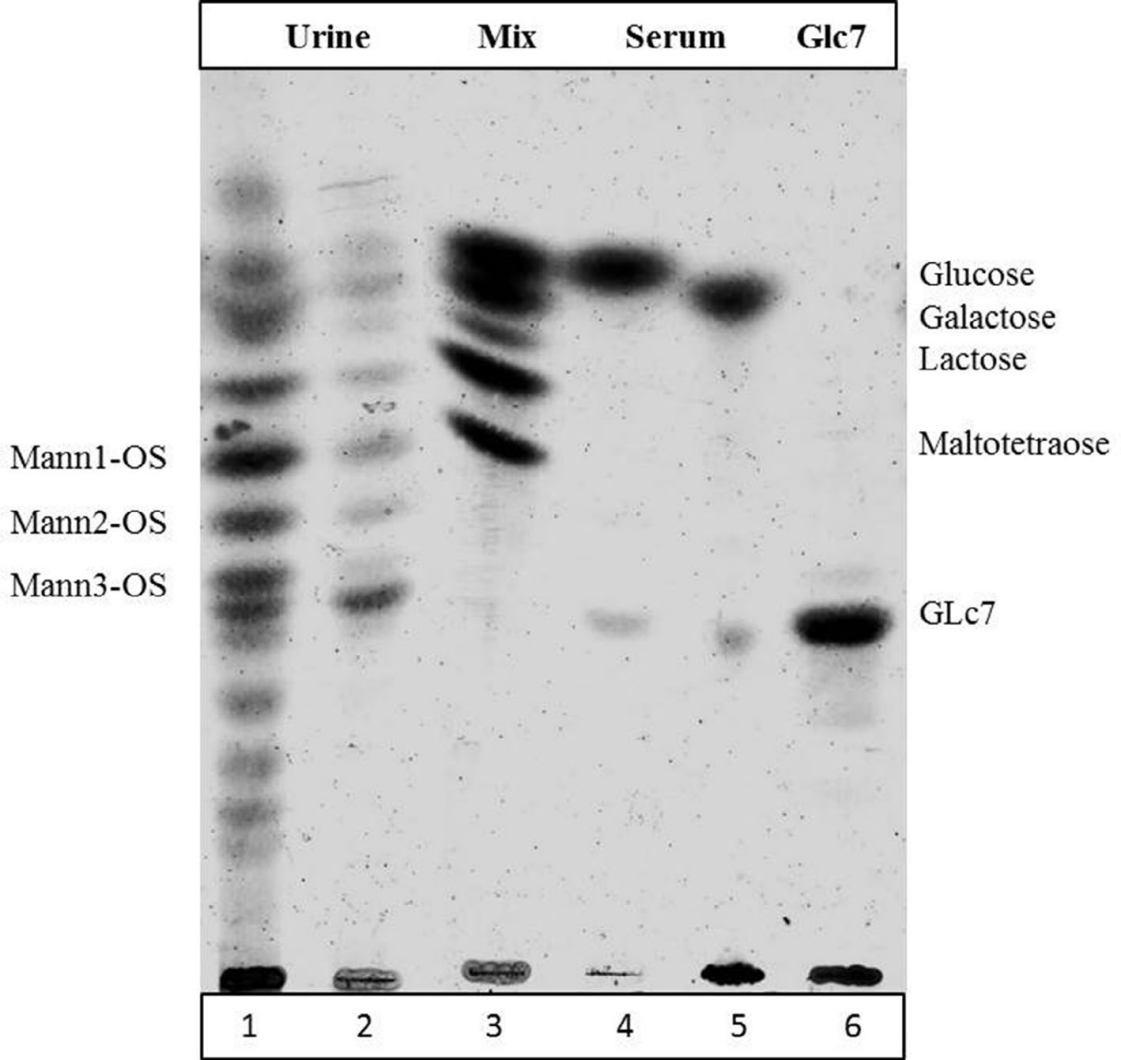
TLC of neutral oligosaccharides in urine (line 1,2) and serum (line 4,5) collected immediately before the first infusion (line 1, 4) and before the eighth infusion (line 2, 5) of VA. Lane 3 contains a standard mixture (Mix) with glucose, galactose, lactose and maltotetraose. Line 6 contains the internal standard (Glc7) used for LC/MS‐MS analyses

A marked reduction of the neutral‐OS in urine samples was observed after 8 weeks of treatment with VA. The bands corresponding to OS containing 2, 3, 4 mannose residues (Mann1‐OS, Mann2‐OS, Mann3‐OS) resulted decreased while the other OS with higher molecular weight were undetectable (Figure 1). The limited sensitivity of the methodology did not allow to show the mannosyl‐OS in serum samples.

**FIGURE 2:**
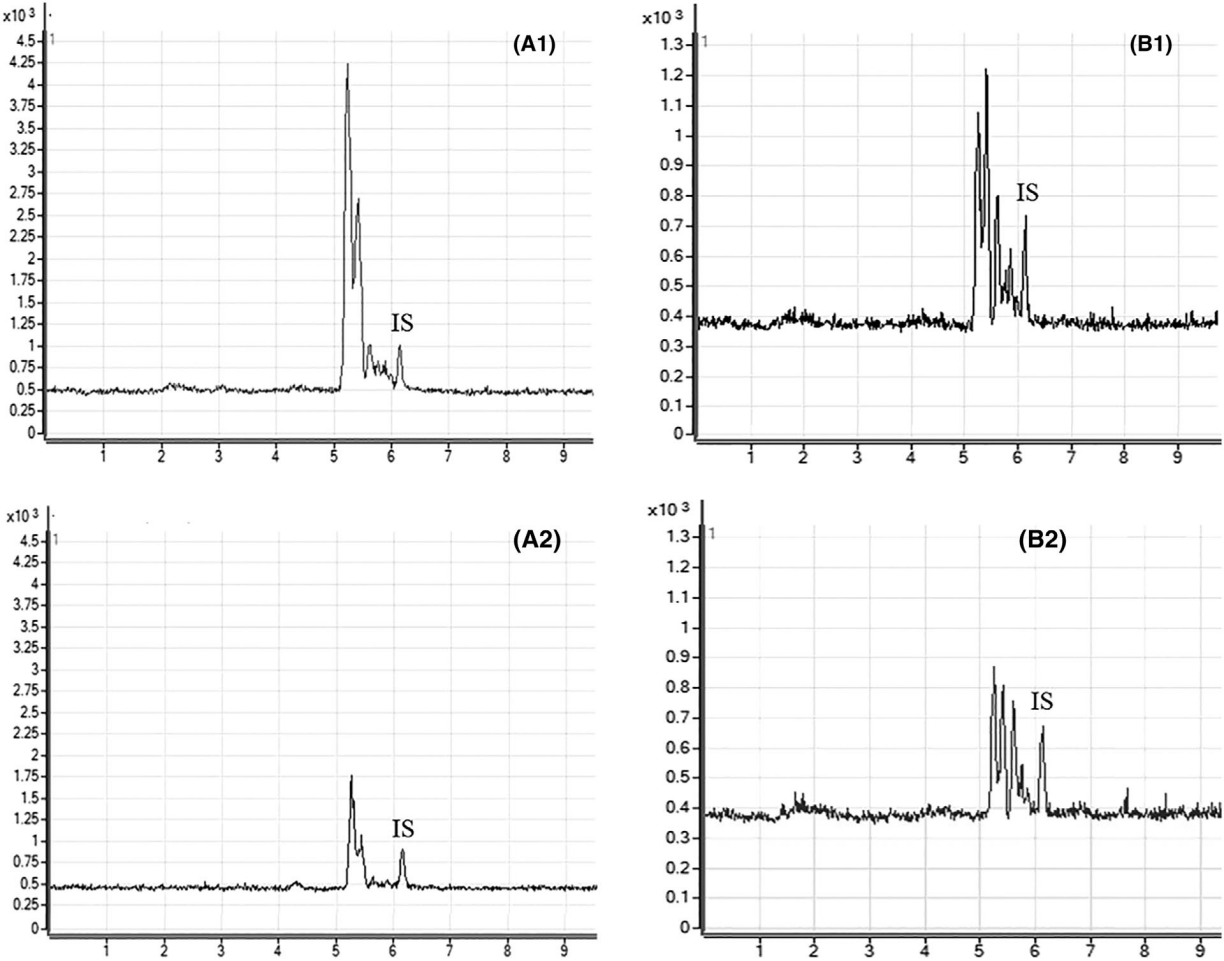
Specific TIC (Total Ion Chromatogram) of mannosyl‐OS in positive mode of urine (A1, A2) and serum (B1, B2) samples collected immediately before the first infusion (A1, B1) and before the eighth infusion of VA (A2, B2)

**FIGURE 3:**
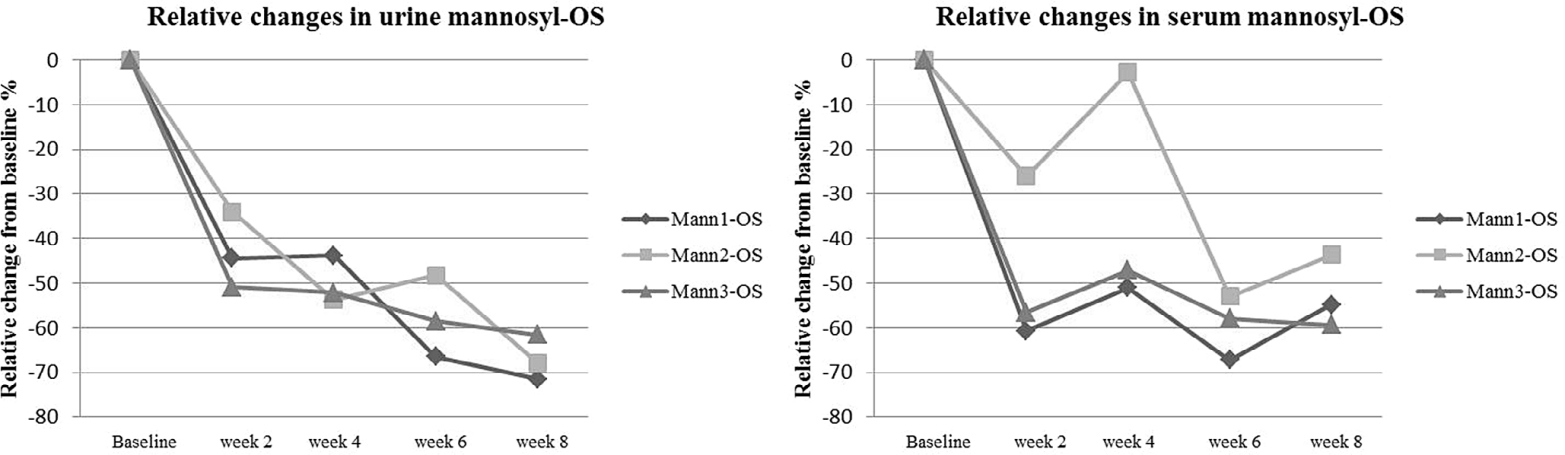
Relative changes of mannosyl‐OS in urine and serum from baseline values to the eighth VA infusion

The analysis by LC‐MS/MS allowed us to monitor the changes of mannosyl‐OS during VA treatment. In Figure 2 were showed the total ion chromatograms (TIC) of the urine and serum samples indicating the presence of three major peaks deduced to be Mann1‐OS, Mann2‐OS, and Mann3‐OS. A reduction of the three peaks was observed after 8 weeks of ERT in urine and serum samples. In particular, the mean relative changes from baseline values at the last observation were −67% in urine and −53% in serum (Figure 3). In urine samples, all the three mannosyl‐OS followed a linear trend of decrease while in serum samples at the fourth week of treatment an increase of Mann2‐OS content was observed.

### 4.3 Alpha‐mannosidasis enzymatic activity

The enzymatic activity of serum alpha‐mannosidasis was monitored before ERT infusions during the 8 weeks of treatment. The activity resulted always upper the normal range (4× normal levels) in the second, fourth, sixth, eighth week of observation with a mean value ± SD of 139 ± 25 nmol/mL/h (mean normal values 35 ± 26).

## 5 DISCUSSION

Alpha mannosidosis is an ultrarare pathology with variable phenotypic manifestations, characterized by the deficiency of lysosomal alpha mannosidase which causes accumulation of mannosyl‐OS since embryonic phase.

In our patient, the early onset of some of the most common features of alpha‐mannosidosis as bone anomalies, hernia, organomegaly and mild coarse face along with the two severe mutations in MAN2 gene lead us to assume a rapid progressive phenotype. For this reason HSCT was planned in order to stabilize or even improve skeletal abnormalities and prevent the cognitive decline and early death. Indeed a stabilization of cognitive functioning and improvement of symptoms were reported by early transplantation.^4^, ^5^, ^6^

Immediately after the diagnosis, the recombinant enzyme VA has been available in Europe for long‐term treatment of non‐neurological manifestations in mild and moderate forms of alpha‐mannosidosis and so the decision of ERT as supporting therapy waiting for HSCT was made.

In this study, we show the very early effects of ERT in a 7 months old patient with alpha mannosidosis. According to our knowledge, this patient is the second case in the world diagnosed so early because of both the rarity of the disease and the complexity of the diagnostic pathway, with an estimated diagnostic delay of about 6 years.^12^ There is no published data on ERT in such young patients as its use below 6 years of age is still under investigation. Currently ERT is not indicated for treatment of neurological manifestations, since its efficacy was not proved.^6^

The aim of the study is to highlight the changes in the biochemical markers associated to alpha‐mannosidosis after treatment with VA.

Our data show that VA treatment is able to reduce the content of mannosyl‐OS monitored both with TLC and LC‐MS/MS. By applying the TLC analysis, a marked reduction of the eight neutral OS isolated from urine was observed before ERT therapy and after 8 weeks. This result led us hypothesize a correlation with the correcting effect of VA.

Furthermore, the LC‐MS/MS technique confirmed the decrease of Mann1‐OS, Mann2‐OS, and Mann3‐OS as showed by TLC. It also allowed monitoring the relative changes of the three different mannosyl‐OS both in urine and serum samples during the 8 weeks of treatment.

The reduction has been observed since the second week of ERT with a decreasing trend up to the end of the second month. An increase of Mann2‐OS at the fourth week was observed in serum probably due to the spontaneous fluctuations in OS concentration.^8^

Other studies have monitored OS during ERT therapy, both in animal models and in alpha‐mannosidosis subjects, showing an OS reduction in different biological samples. The methods used required multiple steps including sample derivatization or combination of different separation techniques.^9^, ^13^, ^14^ In the present study, the direct semi‐quantitative LC‐MS/MS method described by Piraud et al^10^ for the diagnosis of oligosaccharidoses was applied, and for the first time, it was adapted to serum samples in order to monitor ERT treatment. Although a true quantification could not be obtained because of the lack of commercial standard and IS for each compound,^10^ the method is able to highlight variations over time of the different OS both in serum and in urine. In our opinion, this rapid and easy to run method could represent an important tool for monitoring ERT therapy in alpha‐mannosidosis.

An additional important outcome of the study is the enzymatic activity detected in serum 1 week after the first infusion: it was four times higher than the normal values and constant in the following points of observation (fourth, sixth, eighth week). Our results show the presence of circulating enzyme between the infusions, in accordance with the findings of other authors who described the long half‐life of VA compared to other enzymes used for ERT.^13^, ^14^

This study suggests that ERT could be biologically active in this young patient ensuring the presence of significant enzyme activity and reducing stored materials.

## CONFLICT OF INTEREST

L. S., L. Z., L. P., C. M., S. Z., A. D., R. C., T. G., and C. C. declare that they have no conflict of interest.

## INFORMED CONSENT

All procedures followed were in accordance with the ethical standards of the responsible committee on human experimentation (institutional and national) and with the Helsinki Declaration of 1975, as revised in 2000 (5). Informed consent was obtained from all patients for being included in the study. Proof that informed consent was obtained must be available upon request. If doubt exists whether the research was conducted in accordance with the Helsinki Declaration, the authors must explain the rationale for their approach, and demonstrate that the institutional review body explicitly approved the doubtful aspects of the study.
